# Localization-adjusted diagnostic performance and assistance effect of a computer-aided detection system for pneumothorax and consolidation

**DOI:** 10.1038/s41746-022-00658-x

**Published:** 2022-07-30

**Authors:** Sun Yeop Lee, Sangwoo Ha, Min Gyeong Jeon, Hao Li, Hyunju Choi, Hwa Pyung Kim, Ye Ra Choi, Hoseok I, Yeon Joo Jeong, Yoon Ha Park, Hyemin Ahn, Sang Hyup Hong, Hyun Jung Koo, Choong Wook Lee, Min Jae Kim, Yeon Joo Kim, Kyung Won Kim, Jong Mun Choi

**Affiliations:** 1 Department of Medical Artificial Intelligence, Deepnoid, Inc., Seoul, Republic of Korea; 2 Department of Radiology, Seoul Metropolitan Government-Seoul National University Boramae Medical Center, Seoul, Republic of Korea; 3 Department of Radiology, Seoul National University College of Medicine, Seoul, Republic of Korea; 4 Department of Thoracic and Cardiovascular Surgery, Pusan National University School of Medicine, Busan, Republic of Korea; 5 Convergence Medical Institute of Technology, Biomedical Research Institute, Pusan National University Hospital, Busan, Republic of Korea; 6 Department of Radiology and Biomedical Research Institute, Pusan National University Hospital, Busan, Republic of Korea; 7 Department of Internal Medicine, Jawol Health Center, Incheon, Republic of Korea; 8 Department of Radiology and Research Institute of Radiology, Asan Medical Center, University of Ulsan College of Medicine, Seoul, Republic of Korea; 9 Department of Infectious Disease, Asan Medical Center, University of Ulsan College of Medicine, Seoul, Republic of Korea; 10 Department of Respiratory Allergy Medicine, Nowon Eulji Medical Center, Seoul, Republic of Korea

**Keywords:** Respiratory distress syndrome, Radiography, Diagnosis

## Abstract

While many deep-learning-based computer-aided detection systems (CAD) have been developed and commercialized for abnormality detection in chest radiographs (CXR), their ability to localize a target abnormality is rarely reported. Localization accuracy is important in terms of model interpretability, which is crucial in clinical settings. Moreover, diagnostic performances are likely to vary depending on thresholds which define an accurate localization. In a multi-center, stand-alone clinical trial using temporal and external validation datasets of 1,050 CXRs, we evaluated localization accuracy, localization-adjusted discrimination, and calibration of a commercially available deep-learning-based CAD for detecting consolidation and pneumothorax. The CAD achieved image-level AUROC (95% CI) of 0.960 (0.945, 0.975), sensitivity of 0.933 (0.899, 0.959), specificity of 0.948 (0.930, 0.963), dice of 0.691 (0.664, 0.718), moderate calibration for consolidation, and image-level AUROC of 0.978 (0.965, 0.991), sensitivity of 0.956 (0.923, 0.978), specificity of 0.996 (0.989, 0.999), dice of 0.798 (0.770, 0.826), moderate calibration for pneumothorax. Diagnostic performances varied substantially when localization accuracy was accounted for but remained high at the minimum threshold of clinical relevance. In a separate trial for diagnostic impact using 461 CXRs, the causal effect of the CAD assistance on clinicians’ diagnostic performances was estimated. After adjusting for age, sex, dataset, and abnormality type, the CAD improved clinicians’ diagnostic performances on average (OR [95% CI] = 1.73 [1.30, 2.32]; *p* < 0.001), although the effects varied substantially by clinical backgrounds. The CAD was found to have high stand-alone diagnostic performances and may beneficially impact clinicians’ diagnostic performances when used in clinical settings.

## Introduction

Chest radiography is a widely used radiological examination for the evaluation of various pulmonary and thoracic abnormalities. Its low operating cost and low radiation dose compared to other radiological exams make it appropriate for primary examinations in diagnostic or screening settings. While an early, accurate diagnosis is pivotal in prescribing an optimal treatment plan among options such as symptomatic therapy, antibiotics, or surgery, two-dimensional representations on chest radiographs (CXRs) of a three-dimensional thoracic structure presents difficulties in accurate diagnoses. Indeed, diagnostic performance by radiography has been found to have high intra-reader variability and inter-reader variability, especially by training backgrounds and years of experience^1–4^, and has been considered to be suboptimal compared to other examinations such as computerized tomography and ultrasonography^5–10^. Moreover, access to experienced radiologists and trained personnel for radiography are often limited in rural areas or low- and middle-income countries in general^3,11,12^.

A computer-aided detection system (CAD) for abnormality detection in CXRs has been explored as a potential solution to the challenges in CXR-based diagnosis^13–15^. Deep-learning-based algorithms have been developed to detect a single disease such as pneumonia^16^, pneumothorax^17^, tuberculosis^18^, and lung cancer^19^, or multiple diseases at once^20–22^. Several commercially available CADs for CXRs have been shown to achieve diagnostic performance comparable to clinicians when used independently (i.e., stand-alone performance)^23–27^ and improve clinicians’ diagnostic performances (i.e., diagnostic impact)^20,24,28^.

While potential utilities of the CADs seem to be promising, recent reviews have found that multiple crucial aspects of model evaluations have been often neglected, especially for commercial CADs^29–31^. First, few attempts have been taken to evaluate model interpretability, even though regulatory agencies for medical devices require model interpretability measures such as localization accuracy or explanations for algorithmic outputs^32,33^. Localization accuracy serves as one such measure specifically for radiology-related CADs because accurate localization indicates that the model is concentrating on clinically relevant parts, not confounders, on an image to make a decision^34,35^. Inaccurate localization, on the other hand, may induce biases that lead to incorrect human diagnoses (e.g., automation bias)^36^. Despite its relevance, localization accuracy is rarely reported in previous evaluations of CADs. Lesion-level diagnostic performance (i.e., whether the model correctly detects a lesion in an image) has been estimated to account for localization but the definition of accurate lesion localization (e.g., any overlap or 20% overlap) is seldom transparently reported^20,22,28,37,38^.

Second, most assessments of commercialized CADs have been limited to detecting a single type of abnormality (e.g., pneumothorax vs. normal)^39–41^ or any presence of abnormality (i.e., abnormal vs. normal)^42–45^. Efficacy for separately detecting multiple types of abnormalities (e.g., consolidation vs. pneumothorax vs. normal), rather than a simple binary screening for any abnormality, needs to be assessed in one setting to mimic real-world clinical practices. Third, the impact of incorrect CAD predictions in the context of CXR interpretation is unknown as it is rarely reported. Last, few studies on CAD evaluations provide evidence for calibration (i.e., agreement between observed proportions and predicted probabilities)^46^, despite the recommendation by relevant reporting guidelines^47–49^. Calibration is especially important for CADs that aim to support decision-making because poor calibration can be misleading^50^.

In this study, we report findings from two clinical trials for a commercial, deep-learning-based CAD that detects consolidation and pneumothorax in CXRs. We used temporal and external validation datasets to assess localization accuracy, localization-adjusted discrimination (i.e., diagnostic performances by varying definitions of accurate localization), and calibration. Furthermore, in a separate trial for diagnostic impact, the causal effect of the CAD assistance on diagnostic performances for six clinicians of various backgrounds was estimated.

## Results

### The CAD stand-alone trial

Among 2,484 radiographs screened for the stand-alone trial, six with incorrect DICOM file information, 164 with visible artifacts, and 188 duplicates were excluded. The final sample included 1050 radiographs (i.e., one radiograph per individual) as planned by the sample size estimation procedure, and the rest were randomly sampled out (Supplementary Figure 1). All included radiographs were successfully evaluated by the CAD with no operational error and therefore included in the following analyses. There was no missing data in the demographic and clinical variables. The analytic sample consisted of 300 consolidation cases, 250 pneumothorax cases, and 500 normal cases (Table 1). For the consolidation cases, the pneumothorax cases, and the normal cases, the mean age (standard deviation) was 57.1 (14.5), 37.6 (18.2), 48.5 (10.1), and the proportion of males was 69.3%, 86.0%, 55.8%, respectively. The median lesion size (interquartile range) was 60.7 (31.4, 100.7) cm^2^ for consolidation and 69.0 (36.4, 127.2) cm^2^ for pneumothorax. Among the 300 consolidation cases, 78 (26.0%) had more than one lesion annotated, and among the 250 pneumothorax cases, 20 (8.0%) had more than one lesion annotated.

**Table 1:** Sample characteristics for the two trials by data sources and abnormalities.

	The CAD stand-alone trial		
	Total	BMC	PNUH
Total			
* N*	1050	500	550
Age	48.4 (15.4)	48.9 (14.5)	47.9 (16.2)
Male	702 (66.9)	318 (63.6)	384 (69.8)
Lesion size (cm^2^)	64.6 [33.1, 112.1]	63.0 [35.7, 108.2]	66.0 [32.4, 114.4]
More than 1 lesion	98 (17.8)	56 (22.4)	42 (14.0)
Pneumothorax			
* N*	250	100	150
Age	37.6 (18.2)	41.6 (18.2)	35.0 (17.8)
Male	215 (86.0)	81 (81.0)	134 (89.3)
Lesion size (cm^2^)	69.0 [36.4, 127.2]	49.1 [27.3, 103.1]	90.7 [40.7, 142.4]
More than 1 lesion	20 (8.0)	10 (10.0)	10 (6.7)
Consolidation			
* N*	300	150	150
Age	57.1 (14.5)	53.0 (15.7)	61.2 (11.9)
Male	208 (69.3)	106 (70.7)	102 (68.0)
Lesion size (cm^2^)	60.7 [31.4, 100.7]	71.0 [41.0, 118.9]	46.7 [21.5, 90.3]
More than 1 lesion	78 (26.0)	46 (30.7)	32 (21.3)
Normal			
* N*	500	250	250
Age	48.5 (10.1)	49.3 (10.5)	47.7 (9.7)
Male	279 (55.8)	131 (52.4)	148 (59.2)

	The CAD impact trial		
	Total	PadChest	CheXpert
Total			
* N*	461	429	32
Age	53.1 (18.7)	53.8 (18.6)	44.3 (18.1)
Male	258 (56.0)	233 (54.3)	25 (78.1)
Lesion size (cm^2^)	107.38 [60.4, 180.8]	107.4 [57.2, 186.7]	105.5 [62.5, 156.9]
More than 1 lesion	83 (31.8)	80 (34.93)	3 (9.38)
Pneumothorax			
* N*	61	29	32
Age	43.6 (18.0)	42.8 (18.3)	44.3 (18.1)
Male	45 (73.8)	20 (69.0)	25 (78.1)
Lesion size (cm^2^)	95.11 [56.8, 154.4]	80.7 [54.6, 145.5]	105.5 [62.5, 156.9]
More than 1 lesion	4 (6.6)	1 (3.45)	3 (9.38)
Consolidation			
* N*	200		–
Age	61.2 (17.7)		–
Male	137 (68.5)		–
Lesion size (cm^2^)	113.6 [61.3, 198.4]		
More than 1 lesion	79 (39.5)		
Normal			
* N*	200		–
Age	48.0 (16.6)		–
Male	76 (38.0)		–

*CAD* Computer-aided detection system, *BMC* Boramae Medical Center, *PNUH* Pusan National University Hospital, *N* The number of cases.

For consolidation, the CAD achieved image-level AUROC (95% CI) of 0.960 (0.945, 0.975), sensitivity of 0.933 (0.899, 0.959), and specificity of 0.948 (0.930, 0.963) (Table 2). At the lesion-level, sensitivity (95% CI) was 0.912 (0.879, 0.938), and about one false positive lesion per every ten cases (0.104; 109/1050) was found. Dice (interquartile range) was scored at 0.691 (0.664, 0.718). For pneumothorax, the CAD achieved image-level AUC (95% CI) of 0.978 (0.965, 0.991), sensitivity of 0.956 (0.923, 0.978), and specificity of 0.996 (0.989, 0.999). At the lesion-level, sensitivity (95% CI) was 0.941 (0.906, 0.966), and about one false positive lesion per every fifty cases (0.020; 21/1050) was found. Dice (interquartile range) was scored at 0.798 (0.770, 0.826).

**Table 2:** Discrimination performances and localization accuracy of the CAD by abnormalities and hospitals.

	Consolidation			Pneumothorax		
	Pooled	BMC	PNUH	Pooled	BMC	PNUH
**Image-level**						
AUROC (95% CI)	0.960 (0.945, 0.975)	0.986 (0.974, 0.999)	0.932 (0.905, 0.960)	0.978 (0.965, 0.991)	0.965 (0.940, 0.990)	0.987 (0.974, 1.000)
Sensitivity (95% CI)	0.933 (0.899, 0.959)	0.980 (0.943, 0.996)	0.887 (0.825, 0.933)	0.956 (0.923, 0.978)	0.930 (0.861, 0.971)	0.973 (0.933, 0.993)
Specificity (95% CI)	0.948 (0.930, 0.963)	0.986 (0.967, 0.995)	0.915 (0.883, 0.940)	0.996 (0.989, 0.999)	0.998 (0.986, 1.000)	0.995 (0.982, 0.999)
**Lesion-level**						
Sensitivity (95% CI)	0.912 (0.879, 0.938)	0.949 (0.909, 0.976)	0.872 (0.816, 0.916)	0.941 (0.906, 0.966)	0.901 (0.830, 0.949)	0.969 (0.929, 0.990)
False positive lesions per case (false positive lesions/cases)	0.104 (109/1050)	0.078 (39/500)	0.127 (70/550)	0.020 (21/1050)	0.018 (9/500)	0.022 (12/550)
Dice (IQR)	0.691 (0.664, 0.718)	0.766 (0.733, 0.798)	0.613 (0.572, 0.655)	0.798 (0.770, 0.826)	0.747 (0.696, 0.799)	0.833 (0.803, 0.863)

*CAD* Computer-aided detection system, *BMC* Boramae, *PNUH* Pusan National University Hospital, *AUROC* Area under the receiver operating characteristic, *CI* Confidence interval, *IQR* Interquartile range.

After adjusting for age, sex, hospital, abnormality type, and the number of annotated lesions per case, cases with larger lesions were more likely to be correctly diagnosed by the CAD compared to cases with smaller lesions at the image-level (OR [95% CI] = 1.11 [1.07, 1.16]; *p* < 0.001) (Table 3). The association was consistent at the lesion-level (OR [95% CI] = 1.11 [1.07, 1.14]; *p* < 0.001).

**Table 3:** Logistic regression results (image-level) and mixed effects logistic regression results (lesion-level) for associations between lesion characteristics and diagnostic accuracy of the CAD.

	Image-level		Lesion-level	
	OR (95% CI)	*p*-value	OR (95% CI)	*p*-value
Intercept	2.26 (0.33, 16.87)	0.414	2.22 (0.08, 2.72)	0.387
Age	0.98 (0.96, 1.01)	0.267	0.99 (0.99. 1.04)	0.201
Female (vs. male)	0.85 (0.34, 2.23)	0.732	0.83 (0.59, 2.41)	0.619
PNUH (vs. BMC)	0.60 (0.23, 1.50)	0.282	0.82 (0.61, 2.45)	0.586
Pneumothorax (vs. consolidation)	0.84 (0.30, 2.37)	0.736	1.22 (0.39, 1.78)	0.626
More than 1 lesion (vs. a single lesion)	0.55 (0.10, 4.33)	0.513	–	–
Lesion size (cm^2^)	1.11 (1.07, 1.16)	< 0.001	1.11 (1.07, 1.14)	< 0.001

*CAD* Computer-aided detection system, *OR* Odds ratio, *CI* Confidence interval, *PNUH* Pusan National University Hospital, *BMC* Boramae Medical Center.

**Fig. 1:**
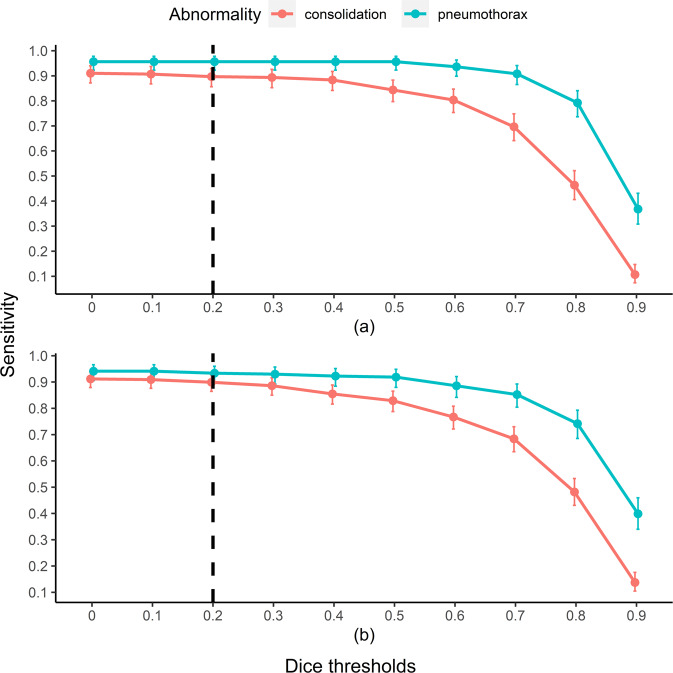
CAD sensitivities by varying dice thresholds in the stand-alone trial. Sensitivities are presented at **a** the image-level and at **b** the lesion-level. The 95% confidence intervals are drawn as error bars at each point. The black dashed line indicates the minimum threshold of clinical relevance (i.e., dice = 0.2). Raw values are presented in Supplementary Table 1. CAD Computer-aided detection system.

As an increasingly stringent definition of accurate localization was applied, sensitivity decreased accordingly at both image-level and lesion-level (Fig. 1; Supplementary Table 1). At dice thresholds of 0, 0.2, 0.4, 0.6, and 0.8, image-level sensitivities were 0.913, 0.897, 0.883, 0.803, 0.463 and lesion sensitivities were 0.912, 0.899, 0.855, 0.767, 0.482 for consolidation. At the same dice thresholds, image-level sensitivities were 0.956, 0.956, 0.956, 0.936, 0.792, and lesion-level sensitivities were 0.941, 0.934, 0.923, 0.886, 0.742 for pneumothorax (Supplementary Figure 2 for examples of predictions at the dice thresholds).

For consolidation, the calibration plot suggested overall moderate calibration (Supplementary Fig. 3). Predicted probabilities were slightly overestimated on average (calibration intercept = −0.249) (Supplementary Table 2). Calibration error (95% CI) was 0.021 (0.013, 0.033) on average and 0.130 (0.068, 0.206). Recalibration in the large (i.e., updating intercept only) was sufficient for recalibration. For pneumothorax, the calibration plot shows moderate calibration, but logistic calibration estimation suggested that the predicted probabilities were also overestimated on average (calibration intercept = −0.584) and overfitted (calibration slope = 0.736). Logistic recalibration (i.e., updating both intercept and slope) was required to improve the calibration performance.

### The CAD impact trial

Among 5,432 radiographs collected for the impact trial, 461 were included as an analytic sample after excluding cases that had artifacts or were random-sampled out. No cases had operational error with the CAD and had missing data in demographic and clinical variables. The analytic sample consisted of 200 consolidation cases, 61 pneumothorax cases, and 200 normal cases (Table 1). For the consolidation cases, the pneumothorax cases, and the normal cases, the mean age (standard deviation) was 61.2 (17.7), 43.6 (18.0), 48.0 (16.6), and the proportion of males was 68.5%, 73.8%, 38.0%, respectively.

**Fig. 2:**
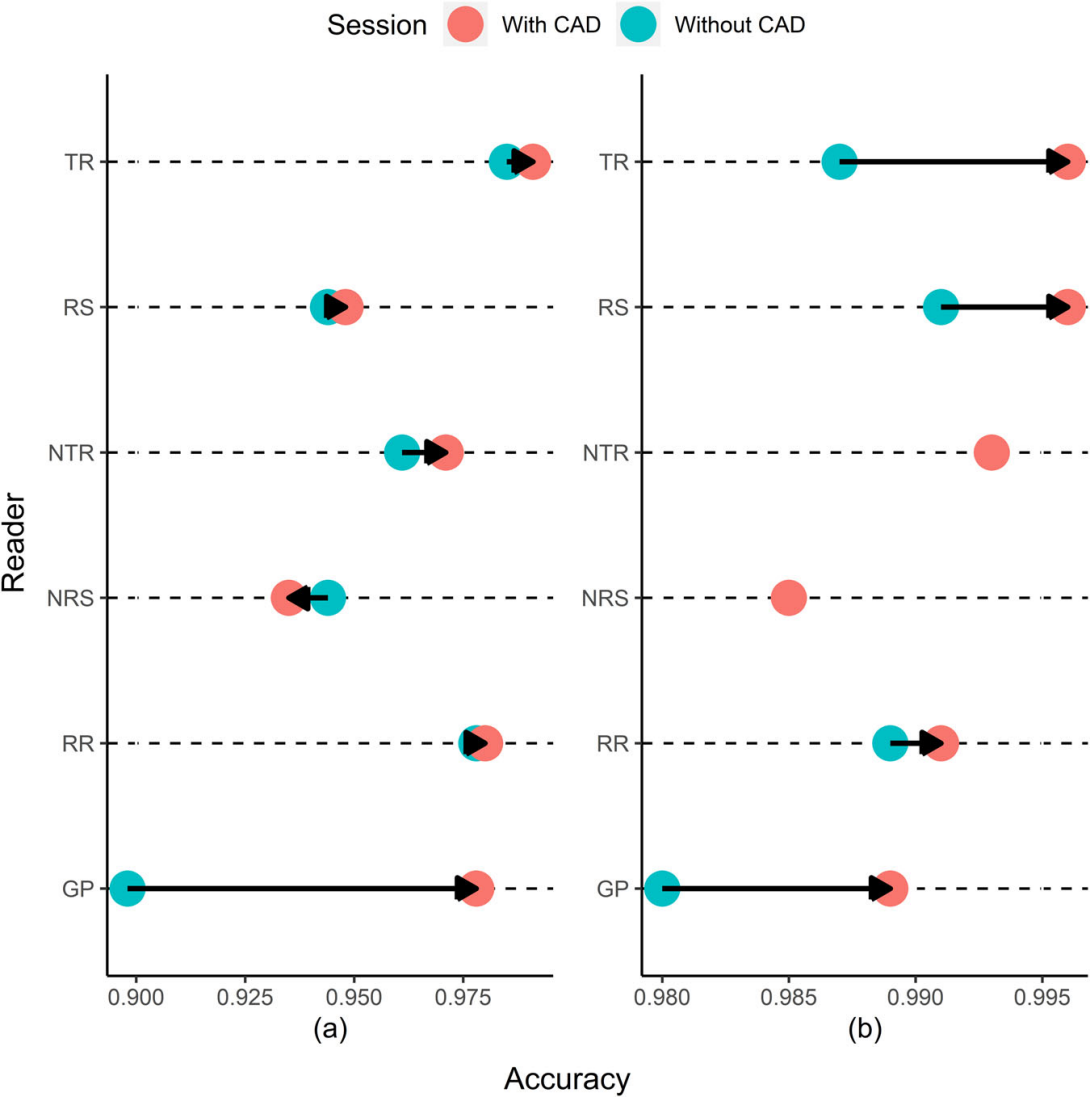
Individual readers’ diagnostic accuracy with and without the CAD assistance in the impact trial. The accuracies are separately presented for **a** consolidation and **b** pneumothorax. The red circle represents accuracy with the CAD assistance, and the blue circle represents accuracy without the CAD assistance. The arrow indicates a directional change in the accuracy when the CAD was used. Raw values are presented in Supplementary Table 3. CAD Computer-aided detection system, TR Thoracic radiologist, RS Respiratory specialist, *NTR* Non-thoracic radiologist, *NRS* Non-respiratory specialist, *RR* Radiology resident, *GP* General practitioner.

The CAD assistance increased six readers’ pooled accuracy (95% CI) from 0.952 (0.943, 0.959) to 0.967 (0.960, 0.974) for consolidation (*p* = 0.001) and from 0.988 (0.983, 0.991) to 0.992 (0.988, 0.995) for pneumothorax (*p* = 0.044) (Table 4). Out of six readers, the CAD assistance increased accuracies for both consolidation and pneumothorax for four readers (i.e., the thoracic radiologist, the respiratory specialist, radiology resident, and general practitioner) (Fig. 2; Supplementary Table 3). For consolidation, unassisted and assisted pooled sensitivities were 0.931 (0.915, 0.945) and 0.980 (0.970, 0.987), respectively (*p* < 0.001), and unassisted and assisted pooled specificities were 0.967 (0.957, 0.976) and 0.958 (0.947, 0.967), respectively (*p* = 0.104). For pneumothorax, unassisted and assisted pooled sensitivities were 0.943 (0.914, 0.964) and 0.956 (0.930, 0.975), respectively (*p* = 0.278), and unassisted and assisted pooled specificities were 0.995 (0.991, 0.997) and 0.997 (0.994, 0.999), respectively (*p* = 0.069).

**Table 4:** Pooled accuracy, sensitivity, and specificity of six readers with and without the CAD assistance.

	Consolidation			Pneumothorax		
	Without CAD assistance	With CAD assistance	*p*-value	Without CAD assistance	With CAD assistance	*p*-value
Accuracy (95% CI)	0.952 (0.943, 0.959)	0.967 (0.960, 0.974)	0.001	0.988 (0.983, 0.991)	0.992 (0.988, 0.995)	0.044
Sensitivity (95% CI)	0.931 (0.915, 0.945)	0.980 (0.970, 0.987)	< 0.001	0.943 (0.914, 0.964)	0.956 (0.930, 0.975)	0.278
Specificity (95% CI)	0.967 (0.957, 0.976)	0.958 (0.947, 0.967)	0.104	0.995 (0.991, 0.997)	0.997 (0.994, 0.999)	0.069

*P*-values are from the comparison between readers’ sensitivity (or specificity) with and without the CAD assistance.

*CAD* Computer-aided detection system, *CI* Confidence interval.

After adjusting for age, sex, dataset, and abnormality type, the odds of an accurate diagnosis with the CAD assistance were 1.73 times (95% CI = [1.30, 2.32]; *p* < 0.001) the odds of an accurate diagnosis without the CAD assistance (Table 5), suggesting that the CAD was able to improve clinicians’ diagnostic performances on average. Readers’ diagnostic performances were improved by a large magnitude when the CAD prediction was correct (OR [95% CI] = 4.84 [3.13, 7.49]; *p* < 0.001). The CAD corrected 69.39 percent (102/147) of all cases where readers alone incorrectly diagnosed (Table 6). The CAD corrected 20 cases among 43 false positives (46.51%) and 82 cases among 104 false negatives (78.85%). Among the unassisted false negatives, the average dice was 0.602 for those corrected and 0.366 for those not affected (d [95% CI] = 0.236 [0.030, 0.442], *p* = 0.029). Incorrect CAD predictions worsened readers’ diagnostic performances (OR [95% CI] = 0.29 [0.17, 0.52]; *p* < 0.001) (Table 5); but only 1.64 percent (43/2619) of all cases for which readers alone correctly diagnosed was misguided by the CAD (Table 6). The CAD misguided 6 cases among 1462 true positives (0.41%) and 37 cases among 1157 true negatives (3.20%).

**Table 5:** Mixed effects logistic regression results for the causal effect of CAD assistance on readers’ diagnostic accuracy.

	Overall		CAD correct		CAD incorrect	
	OR (95% CI)	*p*-value	OR (95% CI)	*p*-value	OR (95% CI)	*p*-value
Intercept	84.18 (32.67, 216.91)	< 0.001	85.79 (30.41, 242.05)	< 0.001	85.00 (9.92, 728.35)	< 0.001
Age	1.00 (0.98, 1.01)	0.548	1.00 (0.99, 1.01)	0.951	0.99 (0.96, 1.02)	0.417
Female (vs. male)	0.79 (0.56, 1.37)	0.349	1.14 (0.67, 1.94)	0.627	0.77 (0.28, 2.09)	0.611
CheXpert (vs. PadChest)	1.40 (0.38, 5.13)	0.608	0.56 (0.13, 2.40)	0.439	2.78 (0.08, 93.14)	0.569
Consolidation (vs. normal)	0.89 (0.52, 1.53)	0.670	0.37 (0.20, 0.69)	0.002	0.67 (0.10, 4.48)	0.677
Pneumothorax (vs. normal)	0.77 (0.28, 2.10)	0.609	1.13 (0.33, 3.91)	0.847	0.04 (0.01, 0.30)	0.001
CAD assistance	1.73 (1.30, 2.32)	< 0.001	4.84 (3.13, 7.49)	< 0.001	0.29 (0.17, 0.52)	< 0.001

*CAD* Computer-aided detection system, *OR* Odds ratio, *CI* Confidence interval

**Table 6:** Details of the CAD effects.

	Values
**Distributions**	
Corrected cases when assisted / incorrect cases when unassisted (%)	102/147 (69.39)
(FP to TN)/FP (%)	20/43 (46.51)
(FN to TP)/FN (%)	82/104 (78.85)
Misguided cases when assisted / originally correct cases when unassisted (%)	43/2619 (1.64)
(TP to FN)/TP (%)	6/1462 (0.41)
(TN to FP)/TN (%)	37/1157 (3.20)
**Dice**	
FN corrected (FN to TP)	0.602
FN not affected (FN to FN)	0.366
Difference (95% CI)	0.236 (0.030, 0.442)

*CAD* Computer-aided detection system, *FP* False positive, *TN* True negative, *FN* False negative, *TP* True positive, *CI* Confidence interval.

## Discussion

In a series of the two trials, we validated the stand-alone diagnostic performances of the CAD for consolidation and pneumothorax, and estimated the causal effect of the CAD assistance on clinicians’ diagnostic performances. In the multi-center, stand-alone trial, the CAD had high AUROC, sensitivity, specificity, moderate calibration at the image-level, and high sensitivity at the lesion-level in the temporal and external validation datasets. Expectedly, the diagnostic performances decreased as we set the threshold for accurate localization more stringently but remained sufficiently high at thresholds well over the minimum level of clinically relevant localization for consolidation and pneumothorax (i.e., dice of 0.2 set by K-MFDS). Using the CAD, the clinicians were able to, on average, improve their diagnostic performances for both consolidation and pneumothorax, although the effect varied substantially by clinical backgrounds.

In temporal and external validation, image-level and lesion-level diagnostic performances of the CAD were higher than previously reported performances of clinicians^5–10^ and comparable to other deep-learning-based algorithms and commercially available CADs in a range of metrics including AUROC, sensitivity, and specificity^21^. Previous published studies on commercial CADs often evaluated their diagnostic performances for consolidation-related diseases^23,27^ or pneumothorax^51^ separately (e.g., pneumothorax vs. non-pneumothorax). Or when targeting multiple types of abnormalities, diagnostic ability for the presence of any abnormality (i.e., abnormal vs. normal), rather than a specific type, was assessed^42,43^. The CAD used in the current trials achieved high diagnostic performances for both consolidation and pneumothorax simultaneously in one setting.

**Fig. 3:**
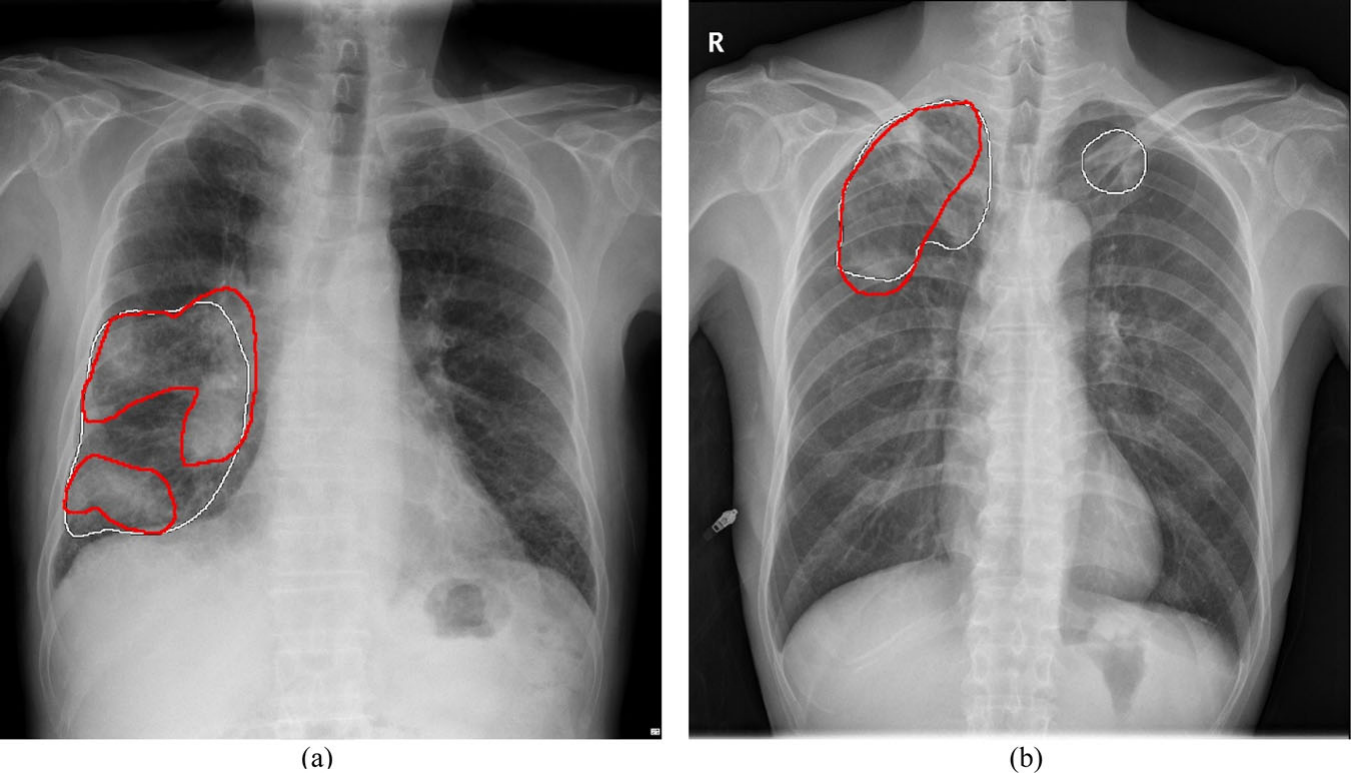
Examples of CAD predictions on chest radiographs. **a** Closely located and diffuse lesions are predicted as a single large lesion, and **b** dense bone structures such as the intersection of the rib and clavicle or sternum were falsely identified as consolidation. CAD Computer-aided detection system.

Previous evidence on CADs has not transparently defined what counted as accurate localization. To gain trust as an interpretable CAD, the CAD should offer not only correct classification, but also clinically interpretable localization^34^. We showed that CAD diagnostic performances can vary widely depending on what is accepted as accurate localization, warranting future studies to assess and transparently report diagnostic performances that accounted for localization accuracy. The present CAD achieved localization accuracy comparable to previously reported clinicians’ localization accuracy^52^ and high diagnostic performances at and beyond the clinically relevant localization threshold determined by K-MFDS. While dice was used as a measure of localization accuracy in this study, other metrics may be more suited for manifestations without clear boundaries, such as diffuse interstitial opacities. Even for consolidation, the CAD tended to clump together separate lesions if they were diffuse and closely located (Fig. 3a). Such CAD prediction may be sufficient as a diagnostic aid, but dice penalizes it. Based on a clinically appropriate standard, localization accuracy of CADs should be routinely assessed as inaccurate lesion localization may lead to automation bias^36^, or worse, correct classification through localization of confounders would erode trust in CAD predictions^53,54^.

Calibration was found to be moderate according to the calibration plots, but the estimation of calibration intercept and slope suggested slight indications of overestimation and overfitting, especially for pneumothorax. Simple recalibration by updating intercept and slope seems to improve performances and should be performed before the clinical implementation of the CAD^55,56^.

The CAD detected lesions larger in size better than smaller lesions, which is an expected (human-like) behavior and consistent with reports on other CADs for CXRs^17,51,52^. The diagnostic ability for small lesions needs improvement because, unlike abnormalities like pleural effusion, small lesions of consolidation or pneumothorax need to be detected as these abnormalities can urgently need clinical attention. For example, if small consolidation is missed in a CXR of a patient presenting fever and cough, they may be erroneously diagnosed as the common cold. An assistance from the CAD localizing consolidation would help physicians appropriately diagnose pneumonia and prescribe antibiotics. The CAD accurately detecting small amount of pneumothorax on a CXR of a patient presenting cough and chest pain would properly guide physicians for chest tube insertion. In addition, for a small number of cases, the CAD falsely identified dense bone structures such as the intersection of the rib and clavicle or sternum as consolidation (Fig. 3b). Future development of the CAD should aim to eliminate such error.

When the clinicians with various backgrounds were assisted by the CAD, their diagnostic performances improved on average, demonstrating the potential clinical impact of the CAD. The overall effect may have been smaller in magnitude for pneumothorax compared to consolidation because the pneumothorax cases from the open datasets appeared to be easier than those encountered in certain clinical settings, such as emergency departments^5,7,42,57,58^; the diagnostic metrics have upper bounds of 1. Importantly, the CAD effects varied substantially by the clinicians’ expertise. The performance gain for the general practitioner was apparent for both consolidation and pneumothorax. For the radiology resident, the thoracic radiologist, and the respiratory specialist, the effects were larger for pneumothorax, while for non-pulmonary specialists, the effects were mixed. The benefits of the CAD use may be maximized in lower-resource settings like community health centers or primary care facilities, where trainee-level or non-radiology clinicians often have responsibilities for reading CXRs due to lack of radiologists, or emergency departments where the burden of diagnostic tasks on pulmonary or respiratory specialists is heavy. Nonetheless, better understanding of a source of heterogeneity in the clinician-specific effects is crucial because not only radiologists, but also other clinicians in primary care, internal medicine, or emergency departments may be potential users of the CAD.

One important observation from the impact trial is that the majority of the CAD effect was from correcting false negatives to true positives, which is reflected in the CAD-induced increase in pooled sensitivity for consolidation. Considering that generally reported sensitivities of clinicians for consolidation and pneumothorax are much lower than those observed for the open datasets used in the impact trial^5–10^, more false negatives are expected in many clinical settings, and potential benefits of using the CAD may be larger. The CAD may be particularly beneficial in screening settings to detect consolidation-presenting diseases such as tuberculosis and pneumonia as they require prompt evaluation and action. Moreover, the localization accuracy of the CAD predictions was higher for the false negatives where the CAD corrected the clinicians’ diagnoses compared to those not affected by the CAD, suggesting that localization accuracy is an important component of the CAD effect on clinicians’ decisions. Another important observation is that while correct CAD predictions had a large positive effect on clinicians’ diagnostic performances, incorrect CAD predictions had a negative effect. In spite of that, only 1.64 percent of correctly diagnosed cases at first were misguided by the CAD, whereas 69.39 percent of incorrectly diagnosed cases at first were corrected by the CAD, indicating that overall, the negative effect was small. However, if the level of diagnostic difficulty was higher in the target population, the CAD stand-alone performance may be lower, and in turn, the assistance effect would be lower. Nonetheless, the magnitude of the CAD effect observed in the study suggests that the effect may withstand such bias.

The unintended consequence of incorrect CAD predictions is expected from any CAD, including the present one, due to automation bias and is probably hard to avoid as long as there is at least one incorrect CAD prediction. Such bias is a reflection more of a human tendency in interpreting any CAD’s decisions rather than mere idiosyncratic characteristics of a specific CAD. Interestingly, a study on a CAD for a different disease using a different modality found the effects of similar magnitudes on the subset of cases with correct or incorrect CAD predictions, suggesting that human may react similarly to automated decisions across various types of CADs^59^. Therefore, one primary aim should be to minimize the total number of incorrect CAD predictions. Another aim could be to amplify the positive effect for cases with correct predictions and to diminish the negative effect for cases with incorrect predictions. One approach is to find optimal CAD outputs and a graphical user interface for the ideal causative mechanism of the CAD effect. For example, rather than simple classification of abnormalities, well-calibrated predicted probability outputs can help clinicians interpret the CAD assistance; CAD predictions with high probability would increase the positive CAD effect and CAD predictions with low probability would mitigate the negative CAD effect.

Our findings have some potential limitations. First, data for both trials were retrospectively collected. While a more rigorous investigation on clinical effectiveness of the CAD requires a prospective trial, such trial may bring risks on patients and require high cost depending on the trial design, and therefore, demands sufficient evidence on utility and safety before being conducted. We believe the stand-alone trial supervised by the regulatory agency and the pilot impact trial now established enough evidence from retrospective data for the CAD to be tested in a prospective trial. Second, the diagnostic performances and the causal effect of the CAD may have limited generalizability. The CAD is currently approved for use in South Korea, and additional studies with data from different populations are needed before using in other countries. It is also possible that diagnostic performances vary within South Korea depending on geographical regions or clinical settings. To increase representativeness, in the stand-alone trial, we included patients from two sources, one large hospital in northern South Korea and the other large hospital in southern South Korea, and in the impact trial, we included data from Spain and the USA. Third, the impact trial utilized the two open datasets. It did not use the stand-alone trial data because it was planned after the stand-alone trial, and the CAD was only recently approved. Nevertheless, both datasets come from reliable sources and have information on demographic variables which are essential in understanding the sample. Fourth, only six clinicians participated in the impact trial, and results may have been different if more clinicians were included. While six is not a small number compared to previous studies^40,57^, it certainly cannot reliably represent potential users of the CAD. We attempted to increase representativeness by recruiting clinicians of various backgrounds including pulmonary specialists, non-pulmonary specialists, and trainee-level clinicians. Fifth, despite the washout period and random ordering of CXRs, recall bias may not have been eliminated completely. We believe that the bias was trivial as the minimum of two-week-long washout period was chosen based on clinical experiences, a sheer number of CXRs to be read, the length adopted in previous studies on CXRs^37,60^, and a guideline on diagnostic validation studies (though not on CXRs)^61^. Furthermore, if a washout period is too long, a period effect may take place, especially in a sequential design used in the impact trial (e.g., gaining experiences or changes in diagnostic criteria over time). Last, the sequential design itself may be prone to recall bias, although we believe the bias is negligible as explained above, and several recent studies have adopted the sequential design^24,57^. A crossover design, in which images are first randomized to two reading sessions then switch after a time interval (“crossover”), is the other common design that reduces recall bias and a period effect, but it does not reflect a clinical practice (i.e., reading half CXRs alone and the other half with CAD assistance).

In two trials, we temporally and externally validated the deep-learning-based CAD for consolidation and pneumothorax by assessing localization-adjusted, stand-alone diagnostic performances and found the beneficial causal effect of the CAD assistance on clinicians’ diagnostic performances. The CAD had high diagnostic performances for consolidation and pneumothorax. Localization accuracy was well over the minimum threshold of clinical relevance, and the diagnostic performances remained high at this threshold when localization accuracy was accounted. The CAD, on average, had positive impacts on radiological judgment on diagnosing consolidation and pneumothorax, but the impacts varied widely depending on readers’ clinical backgrounds. Besides, when the CAD was able to correct the clinicians’ diagnoses, the localization accuracy was higher compared to when the CAD was not able to affect clinicians’ diagnoses. Assessment of location-adjusted diagnostic performances should be a routine practice when evaluating CADs developed to detect abnormalities in medical images. For the present CAD, the impact on treatment strategies and ultimately patient outcomes of the CAD should be assessed, moving beyond the diagnosis impact.

## Methods

The CAD stand-alone trial was a multi-center clinical trial conducted as part of a regulatory approval process for the CAD at the Ministry of Food and Drug Safety in the Republic of Korea (K-MFDS; approved product number: 21-841). The stand-alone trial was approved by the institutional review boards (IRB) of Seoul Metropolitan Government Seoul National University Boramae Medical Center (BMC; IRB number: 10-2020-266) and Pusan National University Hospital (PNUH; IRB number: 2011-016-097). The CAD impact trial was a pilot trial conducted to establish preliminary evidence on diagnostic impact, shortly after the K-MFDS approval of the CAD was granted. The impact trial utilized de-identified, publicly available datasets and did not require an IRB review. As the two trials were designed and carried out separately, and used different datasets and analytic approaches, the methodological details are described separately as below. Details of the present study are reported according to the following reporting guidelines: CONSORT with its AI extension^62–64^, STARD^65,66^, and CLAIM^47^. All data analyses for the two trials were performed in R 4.0.3^67^.

### Computer-aided detection system

The CAD used in the two trials was DEEP:CHEST-XR-03 (version 1.0, Deepnoid, Inc., Seoul, South Korea), a deep-learning-based artificial intelligence software device. With the stand-alone trial results reported in this study, the CAD obtained a regulatory approval for medical use. The CAD was designed to assist physicians in detecting abnormalities (consolidation and pneumothorax) in CXRs of individuals aged between 19 and 73. It takes a DICOM (Digital Imaging and Communications in Medicine) file of a CXR as an input and outputs a predicted probability and a contour around the suspected abnormality lesion (Supplementary Figure 4). It was developed by training approximately 454,000 CXRs from multiple institutions across multiple countries. More details on the device can be found on the official CAD website (www.deepnoid.com/deep-chest).

### The CAD stand-alone trial

For the stand-alone trial, samples were retrospectively collected from BMC (temporal validation) and PNUH (external validation), two university-affiliated research hospitals in the Republic of Korea. We planned to collect a total of 1,050 samples unused in the CAD development, of which 500 were normal cases, 300 were consolidation cases, and 250 were pneumothorax cases. The sample size was calculated with the binomial exact method to allow 80% power at the 5% significance level to precisely estimate the diagnostic performance of the CAD with the expected performances obtained from internal validations during the CAD development process^68,69^. BMC patients were selected from January 1^st^, 2019 to April 30^th^, 2021, whereas PNUH patients were sampled from January 1^st^, 2017 to April 30^th^, 2021. The sampling period was chosen by participating radiologists of each hospital based on the availability of predefined sample size at each hospital. Sampling procedures at the two participating hospitals followed the same guidelines but were conducted independently at each hospital. First, at each hospital, one radiologist reviewed the hospital database to extract patients who were aged between 18 and 74, underwent posteroanterior chest radiography, and were tagged with normal, pneumothorax, or consolidation-related abnormalities. Second, radiographs were sampled in a computer-generated random order and examined to exclude 1) radiographs with artifacts (e.g., central venous catheter, wires from sternotomy or thoracotomy), 2) duplicate radiographs, and 3) radiographs with incorrect patient information in the DICOM files. Third, when the predefined sample size was reached, remaining samples were excluded to prevent unnecessary use of patient data and ensure manageable workloads for participating clinicians.

A total of four radiologists, two per hospital, reviewed CXRs to ensure that the initial diagnoses extracted from the hospital database were correct. Using a web-based medical image annotation software^70^, each radiologist independently labelled, or classified abnormalities for, each radiograph as normal, consolidation, or pneumothorax and annotated, or localized, the region of abnormalities with a freehand drawing tool. If the two radiologists’ labels were discordant, they reached consensus by rereading the CXRs or reviewing CT scans, if available. For localization, all images were adjusted in consensus, using annotations from the radiologist with more years of experience as the base. Consensus reading is a common method of establishing reference standards for abnormalities in CXRs because CT scans cannot be obtained for the retrospective data, and limiting the study population to patients with CT scans reduces generalizability.

A clinical research coordinator at each hospital, blinded to the reference standards, operated the CAD after being trained with a user manual. After the included radiographs were randomly shuffled, the CAD analyzed each radiograph.

Data on patients’ age and biological sex were collected from the hospital database. Lesion size and the number of lesions per case were calculated from the annotations of the reference standards to quantify clinical characteristics of each radiograph.

Sample characteristics were described for the total sample, by data sources, and by abnormality types. Discrimination was measured by abnormality type at the image-level and the lesion-level. For image-level performance, the area under the receiver operating characteristic curve (AUROC), image-level sensitivity and specificity were estimated. For the lesion-level performance, lesion-level sensitivity, and the mean number of false positive lesions per image were estimated. Lesion-level specificity could not be considered as the total number of non-lesion areas did not exist. Probability thresholds for sensitivity and specificity were prespecified based on internal validation results (consolidation: 0.40; pneumothorax: 0.70). As a metric of model interpretability, localization accuracy was measured by dice similarity coefficient^71^, an overlap-based index commonly used in medical image segmentation studies^72,73^. Dice was chosen as most consolidation and pneumothorax cases had visible boundaries on CXRs. Subgroup analyses were performed by hospitals to explore potential heterogeneity in the discrimination performance. Calibration performance was visualized in calibration plots and measured by calibration intercept, calibration slope, maximum calibration error, and average calibration error with 95% confidence intervals based on 500 bootstrap samples. Two recalibration methods were explored: 1) updating the intercept only (i.e., recalibration in the large) and 2) updating the intercept and slope (i.e., logistic recalibration)^74^.

In addition, associations between lesion characteristics and accuracy of the CAD were assessed while adjusting for demographic variables (i.e., sex, age, hospital) and abnormality type. At the image-level, a logistic regression was fitted to regress a binary indicator for an accurate prediction on the number of lesions per case and the lesion size. At the lesion-level, a mixed effects logistic regression was fitted to regress a binary indicator for an accurate prediction on the lesion size with images specified as random effects, adjusting for clustering at the image-level (i.e., a two-level random intercept model). Although a guideline for pneumothorax size calculation suggests a binary classification for simple application in clinical settings^17,75^, calculating lesion size on a continuous scale is simple with a computerized algorithm, and avoiding unnecessary dichotomization would be more methodologically appropriate^76^.

While dice of 0.2 was confirmed by K-MFDS as a minimum level of clinically relevant localization (i.e., localizations with lower dice lack clinical usefulness), image-level sensitivity and lesion-level sensitivity were estimated in a range of dice thresholds to check whether diagnostic performances were robust to various definitions of accurate localizations. Only predictions that exceeded each dice threshold were considered accurate predictions. Unlike sensitivities, specificities could not be considered because dice calculation required a reference standard lesion annotation (i.e., radiographs without abnormalities did not have lesions to annotate).

### The CAD impact trial

For the impact trial, CXRs were collected from two commonly used open datasets with reliable data sources and demographic information, PadChest^77^ and CheXpert^78^. The PadChest dataset consists of all available CXRs at the Hospital Universitario de San Juan, Alicante, Spain from January 2009 to December 2017. The CheXpert consists of CXRs performed between October 2002 and July 2017 in both inpatient and outpatient centers from Stanford Hospital, USA. PadChest was used first to collect CXRs, and CheXpert was added later exclusively to reach the sufficient number of pneumothorax cases. A total of 461 radiographs consisted of 200 normal cases, 200 consolidation cases, and 61 pneumothorax cases. Two radiologists with 15 years of experience, who did not participate in reading sessions, selected radiographs in a computer-generated random order. If publicly available classifications of radiographs were discordant with the radiologists’ classifications, they were excluded. Radiographs with artifacts were also excluded.

Consensus reading was adopted as in the stand-alone trial. The two radiologists who selected radiographs from the open datasets independently labelled the radiographs and reviewed once again to reach consensus for the radiographs with discordant pairs of labels.

For the impact trial, six readers (thoracic radiologist, respiratory specialist, non-thoracic radiologist, non-respiratory specialist, radiology resident, general practitioner) with years of experience (mean = 6.5, range = [1, 12]) were included to cover a wide spectrum of potential users of the CAD. They completed two reading sessions, one without and the other with the CAD assistance, with an interval of two- to four-week-long for each reader. The minimum of a two-week-long interval was determined by the readers as the length long enough to remove recall bias between two sessions. The order of radiographs was randomly shuffled for each reader and each session. At both unassisted and assisted reading sessions, the readers were requested to classify radiographs into consolidation, pneumothorax, or normal cases with no information other than the images. The AiCRO system, a clinical trial image management software, was utilized for viewing the radiographs^79^.

Data on age and biological sex were available in the open datasets. Lesion size and the number of lesions per case were calculated from the radiologists’ annotations to quantify clinical characteristics of each radiograph.

Sample characteristics were described for the total sample, by data sources, and by abnormality types. Pooled accuracy, sensitivity, and specificity of the six readers were compared across the unassisted and assisted reading sessions by performing a mixed-effects logistic regression with radiographs and readers specified as random effects (i.e., a three-level random intercept model). Another mixed-effects logistic regression was performed to estimate the causal effect of CAD assistance on the six readers’ pooled accuracy while adjusting for age, sex, dataset, and abnormality type. The equivalent regression was also separately fitted to the subset of cases for which the CAD prediction was correct and the subset of cases for which the CAD prediction was incorrect to examine the effect of CAD assistance in each subset. The distribution of cases influenced by correct CAD predictions was described as those corrected from false positives to true negatives and from false negatives to true positives. The relationship between localization accuracy of CAD predictions and the CAD effect was assessed by comparing dice coefficients for the false negatives that were corrected by the CAD and the false positives that were not affected by the CAD. For incorrect CAD predictions, distribution of cases influenced was described as those misguided from true positives to false negatives and from true negatives to false positives.

### Reporting summary

Further information on research design is available in the Nature Research Reporting Summary linked to this article.

## Supplementary information

Supplementary Material

Reporting Summary

## Data Availability

Anonymized data generated and analyzed in the data analyses are available upon reasonable request. CXRs used in the study are not publicly available as these are withheld by the hospitals participated in the trials to protect participant privacy.

## Code availability

Code for data analyses is available upon reasonable request. The CAD evaluated in the study is publicly available as a commercial software product (www.deepnoid.com/deep-chest).
