# Supplementary material for: Localization-adjusted diagnostic performance and assistance effect of a computer-aided detection system for pneumothorax and consolidation
Source: NPJ Digit Med. 2022 Jul 30;5:107. doi: 10.1038/s41746-022-00658-x (PMC9339006; doi:10.1038/s41746-022-00658-x)
Supplement: Supplementary file 1 — Supplementary Material [file 41746_2022_658_MOESM1_ESM.docx]

|  | ***Consolidation*** | | ***Pneumothorax*** | |
| --- | --- | --- | --- | --- |
| Dice thresholds | Image-level sensitivity (95% CI) | Lesion-level sensitivity (95% CI) | Image-level sensitivity (95% CI) | Lesion-level sensitivity (95% CI) |
| > 0 | 0.910 (0.872, 0.940) | 0.912 (0.879, 0.938) | 0.956 (0.923, 0.978) | 0.941 (0.906, 0.966) |
| > 0.1 | 0.907 (0.868, 0.937) | 0.909 (0.876, 0.936) | 0.956 (0.923, 0.978) | 0.941 (0.906, 0.966) |
| > 0.2 | 0.897 (0.857, 0.929) | 0.899 (0.864, 0.927) | 0.956 (0.923, 0.978) | 0.934 (0.897, 0.960) |
| > 0.3 | 0.893 (0.853, 0.926) | 0.886 (0.850, 0.916) | 0.956 (0.923, 0.978) | 0.930 (0.893, 0.957) |
| > 0.4 | 0.883 (0.841, 0.917) | 0.855 (0.816, 0.889) | 0.956 (0.923, 0.978) | 0.923 (0.884, 0.951) |
| > 0.5 | 0.843 (0.797, 0.883) | 0.829 (0.788, 0.865) | 0.956 (0.923, 0.978) | 0.919 (0.880, 0.948) |
| > 0.6 | 0.803 (0.754, 0.847) | 0.767 (0.721, 0.808) | 0.936 (0.898, 0.963) | 0.886 (0.842, 0.921) |
| > 0.7 | 0.697 (0.641, 0.748) | 0.684 (0.635, 0.730) | 0.908 (0.865, 0.941) | 0.852 (0.804, 0.892) |
| > 0.8 | 0.463 (0.406, 0.523) | 0.482 (0.431, 0.533) | 0.792 (0.736, 0.841) | 0.742 (0.685, 0.793) |
| > 0.9 | 0.107 (0.074, 0.147) | 0.137 (0.105, 0.176) | 0.368 (0.308, 0.431) | 0.399 (0.340, 0.460) |

Note. Only predictions that exceed each dice threshold were considered as correct prediction.

CAD, computer aided detection device; CI, confidence interval.

**Supplementary Table 1** Image-level and lesion-level sensitivities by varying dice thresholds

Supplementary Table 2 Calibration performances of the CAD by abnormalities

Note. CAD, computer aided detection device; CI, confidence interval.

|  | ***Consolidation*** | | | ***Pneumothorax*** | | |
| --- | --- | --- | --- | --- | --- | --- |
|  | Original | Recalibration in the large | Logistic recalibration | Original | Recalibration in the large | Logistic recalibration |
| Calibration  intercept | -0.249 | -0.008 | 0 | -0.584 | -0.513 | 0 |
| Calibration  slope | 1.108 | 1.108 | 1 | 0.736 | 0.736 | 1 |
| Maximum  calibration  error (95% CI) | 0.130 (0.068, 0.206) | 0.092 (0.051, 0.150) | 0.084 (0.040, 0.154) | 0.273 (0.160, 0.472) | 0.280 (0.115, 0.429) | 0.175 (0.091, 0.376) |
| Average  calibration  error (95% CI) | 0.021 (0.013, 0.033) | 0.018 (0.011, 0.027) | 0.013 (0.004, 0.030) | 0.011 (0.006, 0.020) | 0.012 (0.004, 0.021) | 0.008 (0.003, 0.015) |

Supplementary Table 3 Individual readers’ accuracy, sensitivity, and specificity with and without the CAD assistance in the impact trial

Note. CAD, computer aided detection device; CI, confidence interval.

|  | Accuracy (95% CI) | | Sensitivity (95% CI) | | | Specificity (95% CI) | | |  |
| --- | --- | --- | --- | --- | --- | --- | --- | --- | --- |
| Reader  (years of  experience) | Without CAD assistance | With CAD assistance | | Without CAD assistance | With CAD assistance | | Without CAD assistance | With CAD assistance | |
|  | ***Consolidation*** | | | | | | | | |
| Thoracic  radiologist (11) | 0.985 (0.969, 0.994) | 0.991 (0.978, 0.998) | | 0.985 (0.957, 0.997) | 0.990 (0.964, 0.999) | | 0.985 (0.961, 0.996) | 0.992 (0.973, 0.999) | |
| Respiratory  specialist (7) | 0.944 (0.918, 0.963) | 0.948 (0.924, 0.966) | | 0.985 (0.957, 0.997) | 0.995 (0.972, 0.999) | | 0.912 (0.871, 0.943) | 0.912 (0.871, 0.943) | |
| Non-thoracic  radiologist (5) | 0.961 (0.939, 0.977) | 0.971 (0.952, 0.985) | | 0.985 (0.957, 0.997) | 0.985 (0.957, 0.997) | | 0.943 (0.907, 0.967) | 0.962 (0.931, 0.981) | |
| Non-respiratory  specialist (12) | 0.944 (0.918, 0.963) | 0.935 (0.908, 0.956) | | 0.900 (0.850, 0.938) | 0.985 (0.957, 0.997) | | 0.977 (0.951, 0.992) | 0.897 (0.853, 0.931) | |
| Radiology  resident (3) | 0.978 (0.960, 0.990) | 0.980 (0.963, 0.991) | | 0.955 (0.916, 0.979) | 0.970 (0.936, 0.989) | | 0.996 (0.979, 0.999) | 0.989 (0.967, 0.998) | |
| General  practitioner (1) | 0.898 (0.867, 0.924) | 0.978 (0.960, 0.990) | | 0.775 (0.711, 0.831) | 0.955 (0.916, 0.979) | | 0.992 (0.973, 0.999) | 0.996 (0.979, 0.999) | |
|  | ***Pneumothorax*** | | | | | | | | |
| Thoracic  radiologist (11) | 0.987 (0.972, 0.995) | 0.996 (0.984, 0.999) | | 0.934 (0.841, 0.982) | 0.983 (0.912, 0.999) | | 0.995 (0.982, 0.999) | 0.998 (0.986, 0.999) | |
| Respiratory  specialist (7) | 0.991 (0.78, 0.998) | 0.996 (0.984, 0.999) | | 0.951 (0.863, 0.990) | 0.984 (0.912, 0.999) | | 0.997 (0.986, 0.999) | 0.997 (0.986, 0.999) | |
| Non-thoracic  radiologist (5) | 0.993 (0.981, 0.999) | 0.993 (0.981, 0.999) | | 0.984 (0.912, 0.999) | 0.967 (0.887, 0.996) | | 0.995 (0.982, 0.999) | 0.998 (0.986, 0.999) | |
| Non-respiratory  specialist (12) | 0.985 (0.969, 0.994) | 0.985 (0.969, 0.994) | | 0.918 (0.819, 0.973) | 0.918 (0.819, 0.973) | | 0.995 (0.982, 0.999) | 0.995 (0.982, 0.999) | |
| Radiology  resident (3) | 0.989 (0.975, 0.996) | 0.991 (0.978, 0.998) | | 0.951 (0.863, 0.990) | 0.951 (0.863, 0.990) | | 0.995 (0.982, 0.999) | 0.997 (0.986, 0.999) | |
| General  practitioner (1) | 0.980 (0.963, 0.991) | 0.989 (0.975, 0.996) | | 0.918 (0.819, 0.973) | 0.934 (0.841, 0.982) | | 0.990 (0.975, 0.997) | 0.997 (0.986, 0.999) | |

**Stand-alone trial**

**Impact trial**

2484 radiographs screened

- 1859 from BMC
- 625 from PNUH

1050 radiographs included

- 500 at BMC
- 550 at PNUH

1434 excluded

- 6 incorrect information
- 188 duplicates
- 164 artifacts
- 1076 random-sampled out

5432 radiographs screened

- 5000 from PadChest
- 432 from CheXpert

461 radiographs included

- 429 at PadChest
- 32 at CheXpert

4971 excluded

- 60 artifacts
- 15 unclear labels
- 4896 random-sampled out

Supplementary Figure 1 Sample selection flow diagram. The flows are separately presented for the CAD stand-alone trial and the CAD impact trial.

BMC, Boramae Medical Center; PNUH, Pusan National University Hospital; CAD, computer-assisted detection system.

**Supplementary Figure 2 Examples of predictions at varying dice thresholds in the stand-alone trial.** White contours denote predicted locations for abnormalities. Red contours denote the reference standard locations for consolidation. Blue contours denote the reference standard locations for pneumothorax.

| Consolidation | Pneumothorax |
| --- | --- |
| 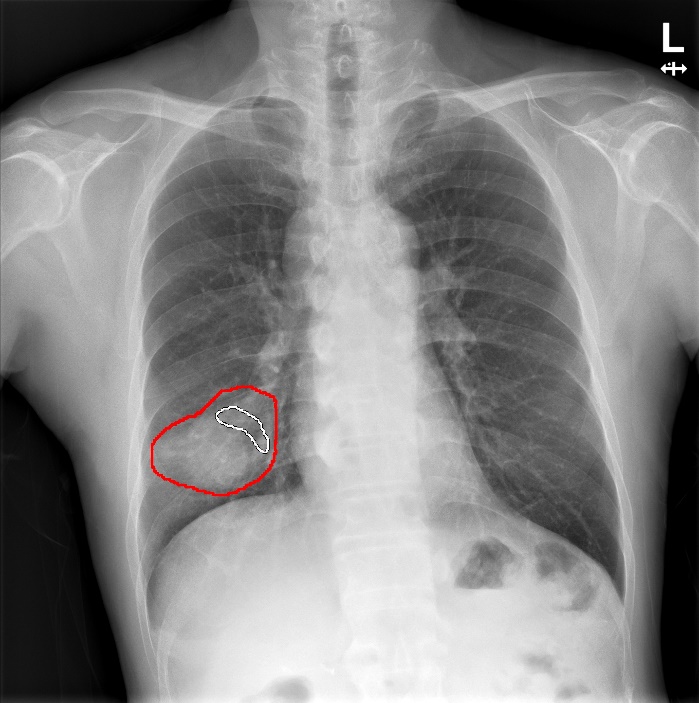 | 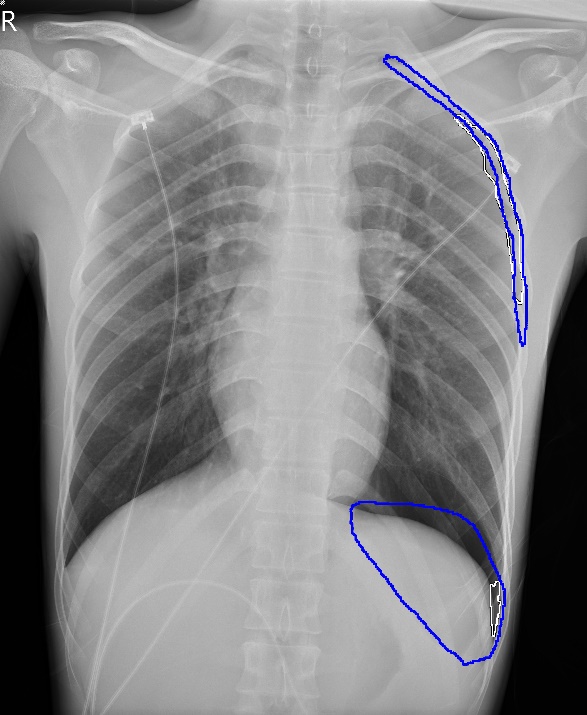 |
| Dice 0.2 | Dice 0.2 |
| 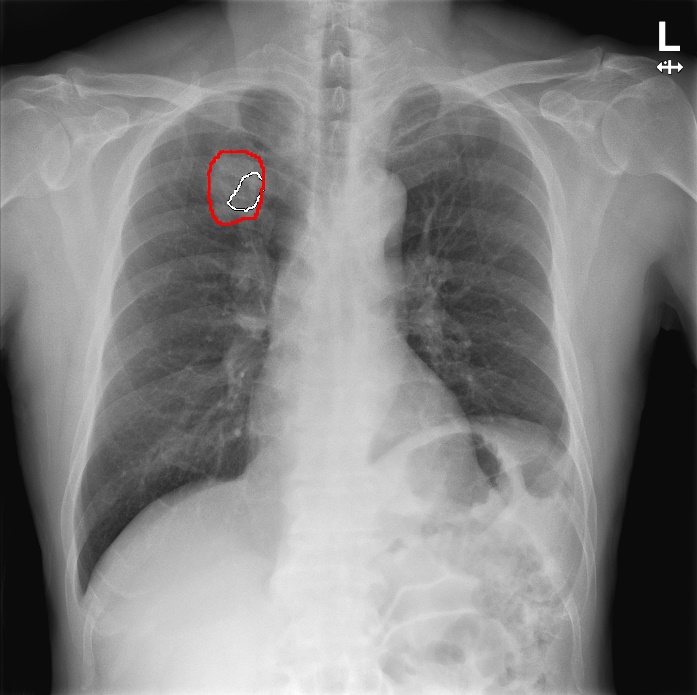 | 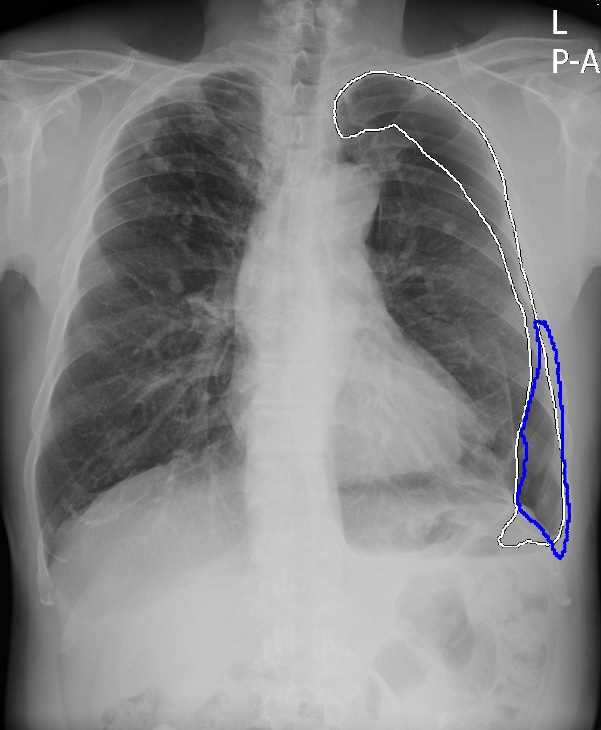 |
| Dice 0.4 | Dice 0.4 |
| 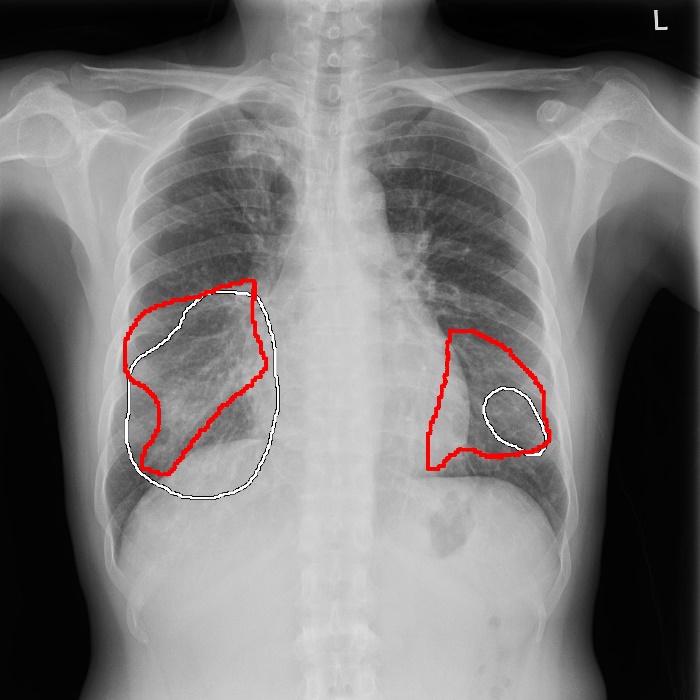 | 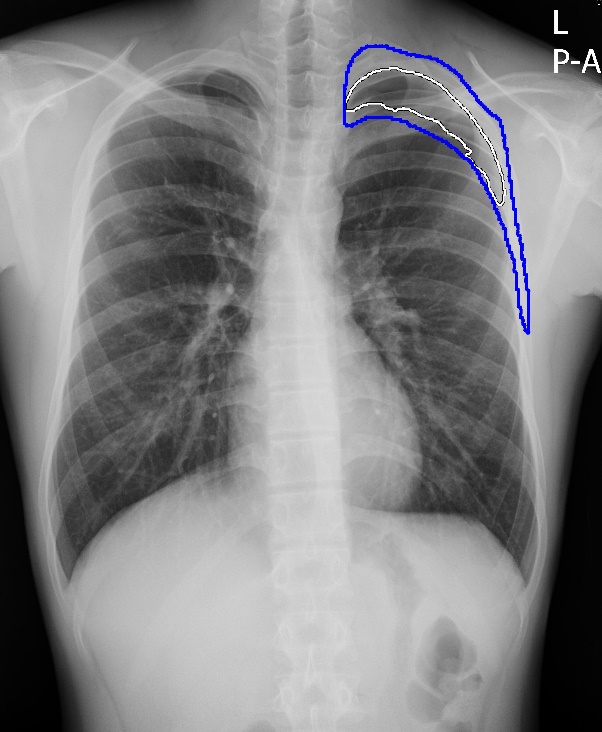 |
| Dice 0.6 | Dice 0.6 |
| 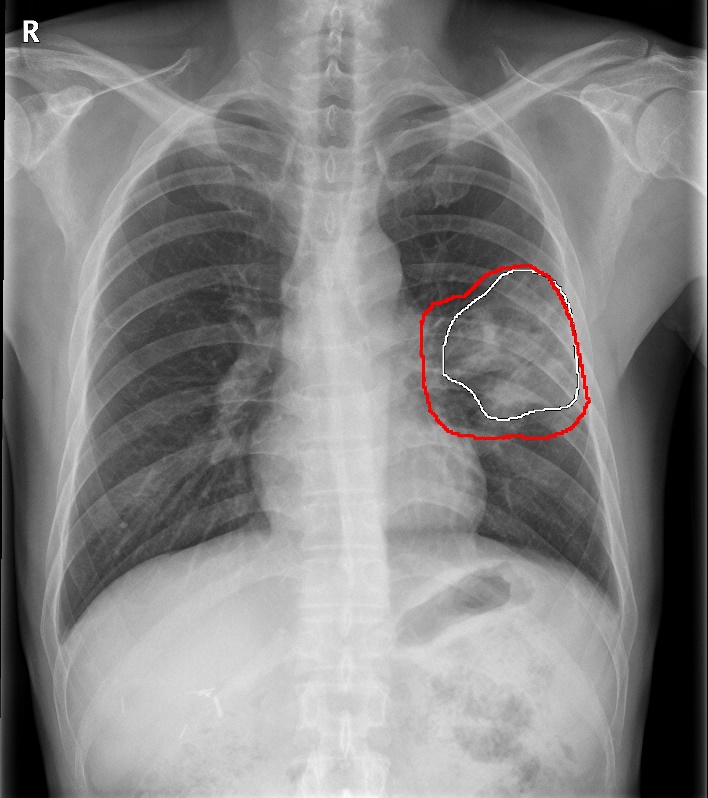 | 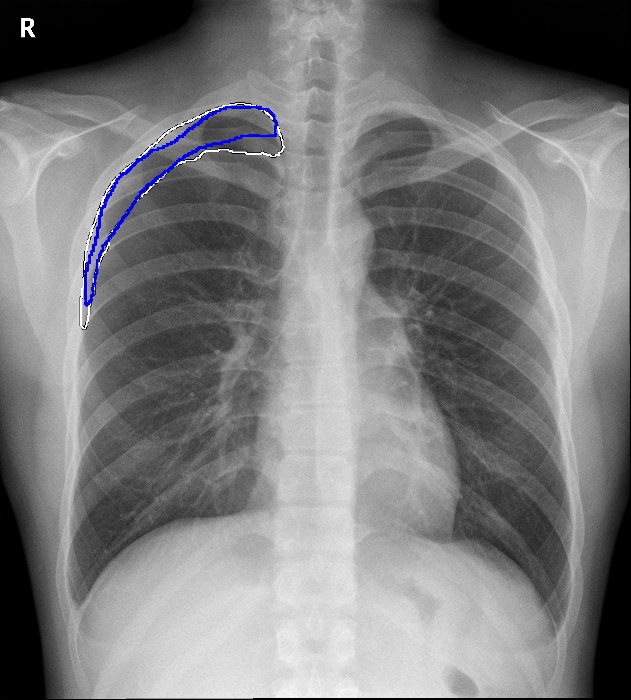 |
| Dice 0.8 | Dice 0.8 |


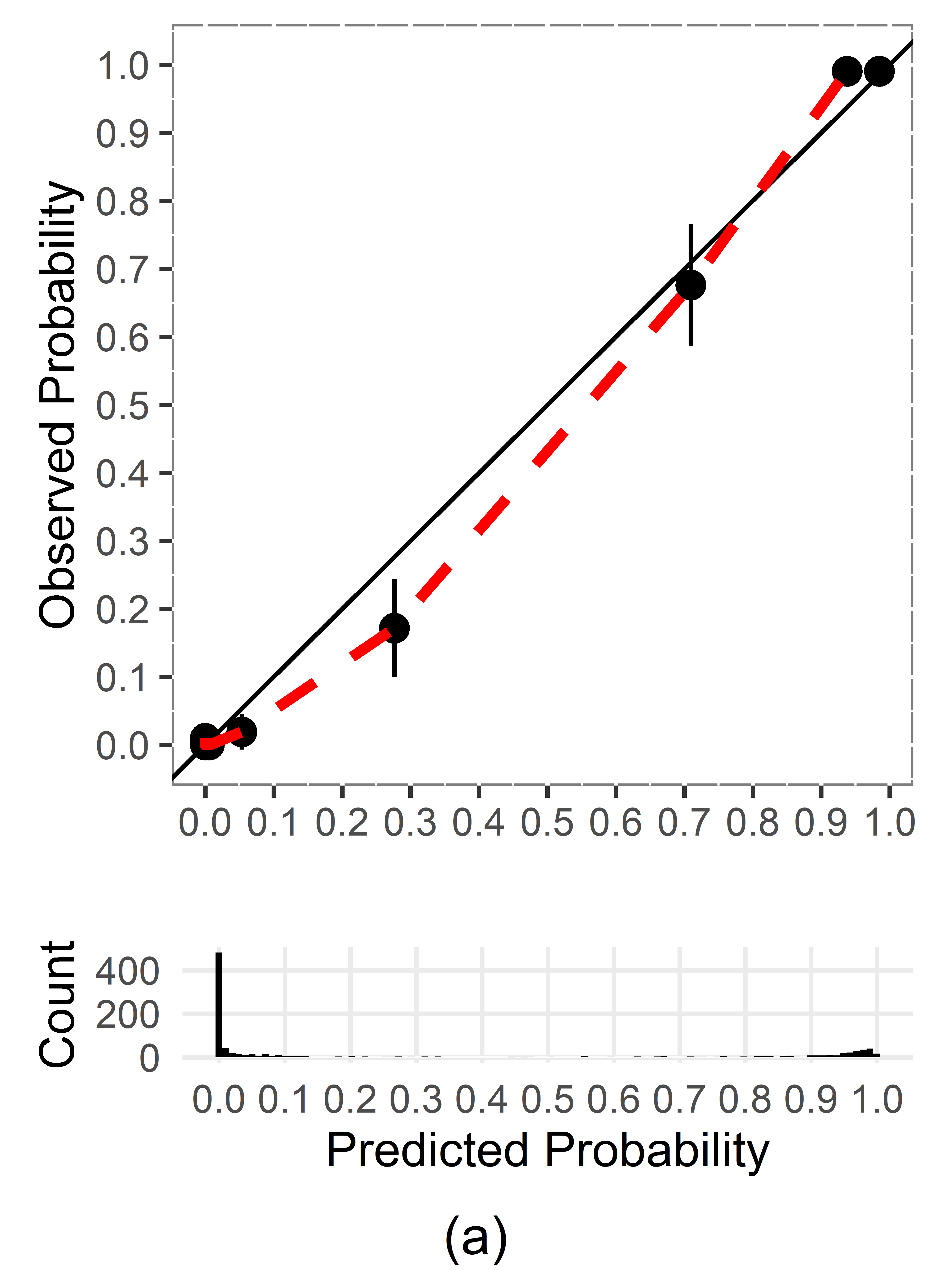

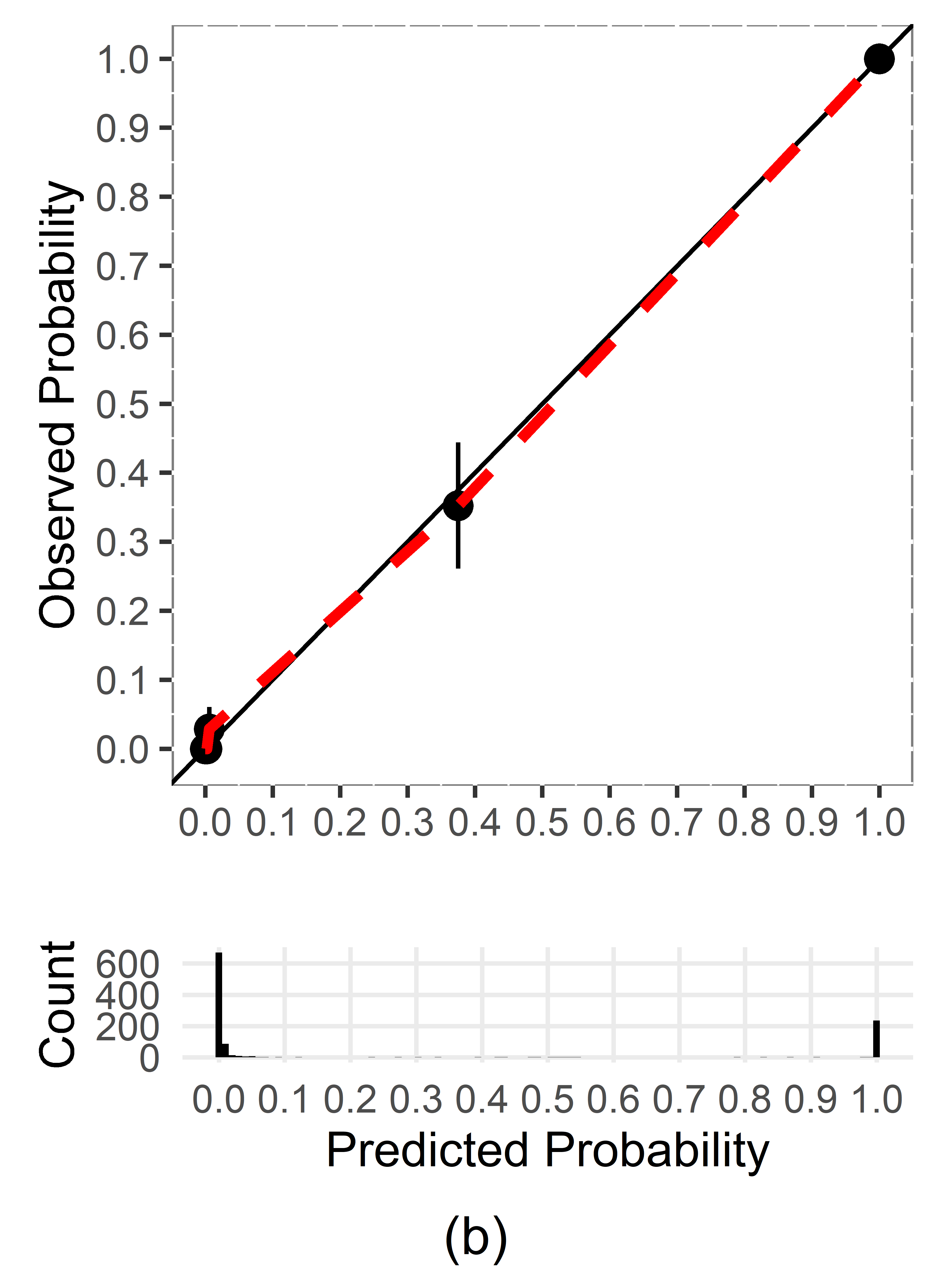


Supplementary Figure 3 Calibration plots and probability distributions in the stand-alone trial. The plots and distributions are separately presented for (a) consolidation and (b) pneumothorax. The black dots in the calibration plots represent deciles of cases grouped by similar predicted probabilities. The 95% confidence intervals are drawn as error bars at each point. The red dashed lines connect the probability deciles.


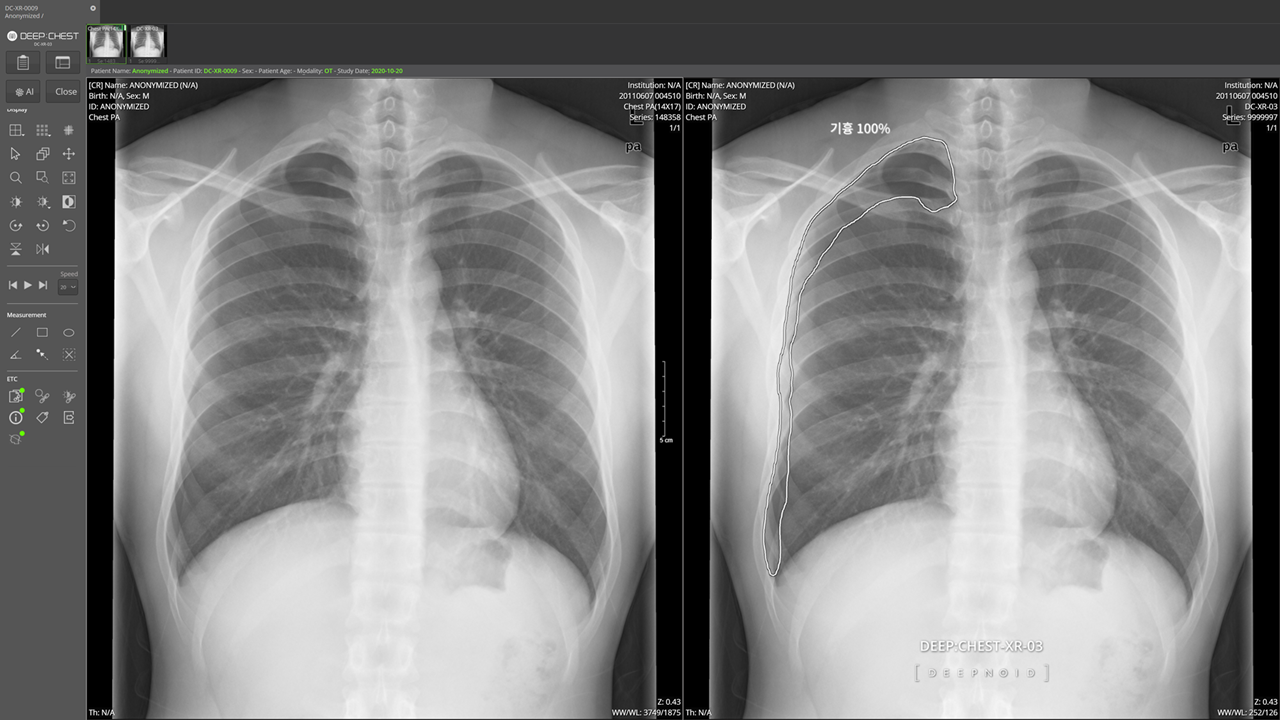


Supplementary Figure 4 The graphic user interface of the CAD (DEEP:CHEST-XR-03). The original image is shown on the left, while the image with the CAD prediction is shown on the right.

CAD, computer-assisted detection system
