# Supplementary material for: Localization-adjusted diagnostic performance and assistance effect of a computer-aided detection system for pneumothorax and consolidation
Source: NPJ Digit Med. 2022 Jul 30;5:107. doi: 10.1038/s41746-022-00658-x (PMC9339006; doi:10.1038/s41746-022-00658-x)
Supplement: Supplementary file 2 — Reporting Summary [file 41746_2022_658_MOESM2_ESM.pdf]

nature portfolio

Corresponding author(s): Jong Mun Choi; NPJDIGITALMED-03437

Last updated by author(s): Apr 1, 2022

Reporting Summary

Nature Portfolio wishes to improve the reproducibility of the work that we publish. This form provides structure for consistency and transparency in reporting. For further information on Nature Portfolio policies, see our Editorial Policies and the Editorial Policy Checklist.

Statistics

For all statistical analyses, confirm that the following items are present in the figure legend, table legend, main text, or Methods section.

n/a	Confirmed	
☐	☒	The exact sample size (n) for each experimental group/condition, given as a discrete number and unit of measurement
☐	☒	A statement on whether measurements were taken from distinct samples or whether the same sample was measured repeatedly
☐	☒	The statistical test(s) used AND whether they are one- or two-sided<br>*Only common tests should be described solely by name; describe more complex techniques in the Methods section.*
☐	☒	A description of all covariates tested
☐	☒	A description of any assumptions or corrections, such as tests of normality and adjustment for multiple comparisons
☐	☒	A full description of the statistical parameters including central tendency (e.g. means) or other basic estimates (e.g. regression coefficient) AND variation (e.g. standard deviation) or associated estimates of uncertainty (e.g. confidence intervals)
☐	☒	For null hypothesis testing, the test statistic (e.g. F, t, r) with confidence intervals, effect sizes, degrees of freedom and P value noted<br>*Give P values as exact values whenever suitable.*
☐	☒	For Bayesian analysis, information on the choice of priors and Markov chain Monte Carlo settings
☐	☒	For hierarchical and complex designs, identification of the appropriate level for tests and full reporting of outcomes
☐	☒	Estimates of effect sizes (e.g. Cohen's d, Pearson's r), indicating how they were calculated

Our web collection on statistics for biologists contains articles on many of the points above.

Software and code

Policy information about availability of computer code

Data collection	No software was used.
Data analysis	Code for data analyses is available upon reasonable request. The CAD evaluated in the study is publicly available as a commercial software product (https://en.deepnoid.com/deepchest).

For manuscripts utilizing custom algorithms or software that are central to the research but not yet described in published literature, software must be made available to editors and reviewers. We strongly encourage code deposition in a community repository (e.g. GitHub). See the Nature Portfolio guidelines for submitting code & software for further information.

Data

Policy information about availability of data

All manuscripts must include a data availability statement. This statement should provide the following information, where applicable:

- Accession codes, unique identifiers, or web links for publicly available datasets
- A description of any restrictions on data availability
- For clinical datasets or third party data, please ensure that the statement adheres to our policy

Anonymized data generated and analyzed in the data analyses are available upon reasonable request. CXRs used in the study are not publicly available as these are withheld by the hospitals participated in the trials to protect participant privacy.

Field-specific reporting

Please select the one below that is the best fit for your research. If you are not sure, read the appropriate sections before making your selection.

☐ Life sciences ☒ Behavioural & social sciences ☐ Ecological, evolutionary & environmental sciences

For a reference copy of the document with all sections, see nature.com/documents/nr-reporting-summary-flat.pdf

Behavioural & social sciences study design

All studies must disclose on these points even when the disclosure is negative.

Study description	The stand-alone trial was a retrospective, diagnostic study.<br><br>The impact trial was a retrospective, diagnostic, multi-reader, multi-case study.
Research sample	For the stand-alone trial, samples were retrospectively collected from Boramae Medical Center and Pusan National University Hospital, two university-affiliated research hospitals in the Republic of Korea.<br><br>For the impact trial, the PadChest dataset consists of all the available chest radiographs at the Hospital Universitario de San Juan, Alicante, Spain from January 2009 to December 2017. The CheXpert consists of chest radiographs performed between October 2002 and July 2017 in both inpatient and outpatient centers from Stanford Hospital, USA.
Sampling strategy	For the stand-alone trial, we planned to collect a total of 1,050 samples unused in the software development, of which 500 were normal cases, 300 were consolidation cases, and 250 were pneumothorax cases. The sample size was calculated with the binomial exact method to allow 80% power at the 5% significance level to precisely estimate the diagnostic performance of the software with the expected performances obtained from internal validations during the software development process.<br><br>For the impact trial, the PadChest dataset consists of all the available chest radiographs at the Hospital Universitario de San Juan, Alicante, Spain from January 2009 to December 2017. The CheXpert consists of chest radiographs performed between October 2002 and July 2017 in both inpatient and outpatient centers from Stanford Hospital, USA. PadChest was used first to collect CXRs, and CheXpert was added later exclusively to reach the sufficient number of pneumothorax cases. A total of 461 radiographs consisted of 200 normal cases, 200 consolidation cases, and 61 pneumothorax cases.
Data collection	For the stand-alone trial, sampling procedures at the two participating hospitals followed the same guidelines but were conducted independently at each hospital. First, at each hospital, one radiologist reviewed the hospital database to extract patients who were aged between 18 and 74, underwent posteroanterior chest radiography, and were tagged with normal, pneumothorax, or consolidation-related abnormalities. Second, radiographs were sampled in a computer-generated random order and examined to exclude 1) radiographs with artifacts (e.g., central venous catheter, wires from sternotomy or thoracotomy), 2) duplicate radiographs, and 3) radiographs with incorrect patient information in the DICOM files. Third, when the predefined sample size was reached, remaining samples were excluded to prevent unnecessary use of patient data and ensure manageable workloads for participating clinicians.<br><br>For the impact trial, chest radiographs were collected from two commonly used open datasets with reliable data sources and demographic information, PadChest and CheXpert. Two radiologists with 15 years of experience, who did not participate in reading sessions, selected radiographs in a computer-generated random order.
Timing	For the stand-alone trial, Boramae Medical Center patients were selected from January 1st, 2019 to April 30th, 2021, whereas Pusan National University Hospital patients were sampled from January 1st, 2017 to April 30th, 2021. The sampling period was chosen by participating radiologists of each hospital based on the availability of predefined sample size at each hospital.<br><br>For the impact trial, the PadChest dataset consists of all the available chest radiographs at the Hospital Universitario de San Juan, Alicante, Spain from January 2009 to December 2017. The CheXpert consists of chest radiographs performed between October 2002 and July 2017 in both inpatient and outpatient centers from Stanford Hospital, USA.
Data exclusions	For the stand-alone trial, radiographs with artifacts (e.g., central venous catheter, wires from sternotomy or thoracotomy), duplicate radiographs, and radiographs with incorrect patient information in the DICOM files were excluded. When the predefined sample size was reached, remaining samples were excluded to prevent unnecessary use of patient data and ensure manageable workloads for participating clinicians.<br><br>For the impact trial, if publicly available classifications of radiographs were discordant with the radiologists' classifications, they were excluded. Radiographs with artifacts were also excluded. When the predefined sample size was reached, remaining samples were excluded to prevent unnecessary use of patient data and ensure manageable workloads for participating clinicians.
Non-participation	No participants dropped out of the study.
Randomization	Randomization was not performed in the study.

Reporting for specific materials, systems and methods

We require information from authors about some types of materials, experimental systems and methods used in many studies. Here, indicate whether each material, system or method listed is relevant to your study. If you are not sure if a list item applies to your research, read the appropriate section before selecting a response.

Materials & experimental systems

n/a	Involved in the study
☒	☐ Antibodies
☒	☐ Eukaryotic cell lines
☒	☐ Palaeontology and archaeology
☒	☐ Animals and other organisms
☒	☐ Human research participants
☐	☒ Clinical data
☒	☐ Dual use research of concern

Methods

n/a	Involved in the study
☒	☐ ChIP-seq
☒	☐ Flow cytometry
☒	☐ MRI-based neuroimaging

Clinical data

Policy information about clinical studies

All manuscripts should comply with the ICMJE guidelines for publication of clinical research and a completed CONSORT checklist must be included with all submissions.

Clinical trial registration

The trials are not registered in any public clinical trial database, but they only used retrospective data and were supervised by the MFDS (FDA-equivalent in South Korea) and IRBs.

Study protocol

The trials are not registered in any public clinical trial database, but they were supervised by the MFDS (FDA-equivalent in South Korea). Therefore, there is no study protocol that is publicly available. However, the manuscript describes in detail about the study procedures.

Data collection

For the stand-alone trial, samples were retrospectively collected from Boramae Medical Center and Pusan National University Hospital, two university-affiliated research hospitals in the Republic of Korea. Boramae Medical Center patients were selected from January 1st, 2019 to April 30th, 2021, whereas Pusan National University Hospital patients were sampled from January 1st, 2017 to April 30th, 2021.

For the impact trial, chest radiographs were collected from two commonly used open datasets with reliable data source and demographic information, PadChest and CheXpert. The PadChest dataset consists of all the available chest radiographs at the Hospital Universitario de San Juan, Alicante, Spain from January 2009 to December 2017. The CheXpert consists of chest radiographs performed between October 2002 and July 2017 in both inpatient and outpatient centers from Stanford Hospital, USA.

Outcomes

For the stand-alone trial, a total of four radiologists, two per hospital, reviewed chest radiographs to ensure that the initial diagnoses extracted from the hospital database were correct. Using a web-based medical image annotation software, each radiologist independently labelled (i.e., classified abnormalities for) each radiograph as normal, consolidation, or pneumothorax and annotated (i.e., localized) the region of abnormalities with a freehand drawing tool. If the two radiologists' labels were discordant, they reached consensus by rereading the chest radiographs or reviewing CT scans, if available. For localization, all images were adjusted in consensus, using annotations of the radiologist with more years of experience as the base.

For the impact trial, the two radiologists who selected radiographs from the open datasets independently labelled the radiographs and reviewed once again to reach consensus for the radiographs with discordant pairs of labels.

Consensus reading is a common method of establishing reference standards for abnormalities in chest radiographs because CT scans cannot be obtained for the retrospective data, and limiting the study population to patients with CT scans reduces generalizability.
